# Clinical Effects of a Natural Extract of Urinary Human Menopausal Gonadotrophin in Normogonadotropic Infertile Patients

**DOI:** 10.1155/2013/135258

**Published:** 2013-03-24

**Authors:** Rui Hua, Lan Ma, Hong Li

**Affiliations:** Center for Reproductive Medicine, Department of Obstetrics and Gynecology, Nanfang Hospital, Southern Medical University, Guangzhou 510515, China

## Abstract

Purified human menopausal gonadotropin (HMG) is a natural product extracted from the urine of postmenopausal women that contains pituitary follicle-stimulating hormone (FSH), luteinizing hormone (LH), and a small amount of human chorionic gonadotropin (HCG). Here we retrospectively conducted a clinical pharmaceutical study on a cohort of normogonadotropic infertile patients addressed to long GnRH-agonist protocol with serum LH concentration ranging from 0.5 IU/L to 1.5 IU/L during the midfollicle phase, aiming at evaluating the effects of purified HMG supplementation during ovarian stimulation. There was no significant difference in either the basic clinical features of the patients or the pregnancy rate (71.4% versus 66.3%, *P* > 0.05) or other related indicators of pregnancy outcome. However, there was a higher level of serum oestradiol (E2) on the day of human chorionic gonadotropin (HCG) (1999.10 ± 860.50 IU/L versus 2883.29 ± 1427.382 IU/L, *P* = 0.000) but lower fertilization rate (89.1% versus 69.6%, *P* < 0.000) in patients getting HMG supplementation and a higher risk of developing ovarian hyperstimulation syndrome (OHSS). We suppose that exogenous LH supplementation is not needed when serum LH concentration of the midfollicle phase is around 0.5–1.5 IU/L during the long GnRH-agonist protocol. Adding exogenous HMG may decrease the fertilization rate and increase the risk of developing OHSS.

## 1. Introduction

Gonadotropins (or glycoprotein hormones) are protein hormones secreted by gonadotrope cells from the anterior pituitary of vertebrates, including the mammalian hormones FSH and LH. These hormones are central to the complex endocrine system that regulates sexual development and reproductive function. LH and FSH are heterodimers consisting of two peptide chains, an alpha chain and a beta chain, and they share nearly identical alpha chains (about 100 amino acids long), whereas the beta chain provides specificity for receptor interactions. Purified human menopausal gonadotropin (HMG, Urofollitropin for Injection-Livzon Pharmaceutical Factory) is an active substance for the treatment of fertility disturbances which is widely used in assisted reproductive technology. It is a product extracted through a sterile preparation of placental glucoprotein from the urine of postmenopausal women and is purified by proprietary chromatography. The purity is greater than 98% as determined by RP-HPLC, anion-exchange FPLC, and reducing and nonreducing SDS-PAGE silver stained gel. Every 75 IU of HMG contains activities of 75 IU of FSH, 75 IU of LH, and a small amount of human chorionic gonadotropin (HCG). The protein quantification was carried out by two independent methods, including UV spectroscopy at 280 nm and RF-HPLC controlled by a standard solution of HMG.

The importance of LH and FSH in human reproduction is explained in the two-cell two-gonadotropin theory [1–4], which demonstrates their separate but complementary roles in regulating follicular growth and ovulation. As a simplified generalization, LH stimulated the theca cells of the ovaries to produce testosterone, whereas FSH stimulates the granulose cells of ovarian follicles to produce cytochrome P450 aromatase which converts the androgens to estrogens. 

Currently, the most commonly used protocol in assisted reproductive technology includes daily injections of recombined human FSH (rFSH) following the pituitary downregulation achieved by GnRH analogues, which plays an important role in preventing a premature LH surge and early ovulation but is also the reason of endogenous LH suppression and deficiency. According to the threshold theory of LH in ovarian function [3, 5, 6], the ovarian follicle requires a minimal amount of LH for steroidogenesis (<1% of receptors attached by LH), however there is still no confirmed definition of the “minimal amount of LH” needed in follicle development. There was a retrospective analysis in the year of 2000 that found that normogonadotrophic patients undergoing long GnRH-agonist protocol treatment were five times more likely to suffer from early pregnancy loss when midfollicular phase (during stimulation days 6–8) LH serum concentrations were below 0.5 IU/L [7]. However, although LH is essential for estrogen synthesis and maintenance of follicular dominance, there is both laboratory and clinical evidence that excessive stimulation of the ovaries by LH may adversely affect the development of human preovulatory follicles [8]. Depending on the stage of development, follicles exposed to inappropriately high concentrations of LH may enter atresia or become prematurely luteinized and oocyte development may be compromised. Therefore, the optimal amount of LH and the best timing of LH supplementation during follicle development need to be further explored.

Here we retrospectively conducted a clinical pharmaceutical study on a cohort of normogonadotropic infertile patients addressed to long GnRH-agonist protocol with serum LH concentration ranging from 0.5 IU/L to 1.5 IU/L on stimulation days 6–8 to investigate the effect of purified HMG supplement during ovarian stimulation.

## 2. Materials and Methods

### 2.1. Patients

 122 normogonadotropic infertile patients were recruited, who were undergoing their first IVF/ICSI cycle in the Center of Nanfang Hospital, Southern Medical University, from November 2010 to March 2012. Main inclusion criteria were indication for treatment with IVF or ICSI; normal menstrual rhythm (25–34 days); age between 20 and 39 years; body mass index (BMI) between 18 and 25 kg/m^2^; baseline FSH < 10 IU/L; midfollicular phase serum LH level on days 6–8 of ovarian stimulation ranged between 0.5–1.0 IU/L. Main exclusion criteria were history of recurrent pregnancy loss; polycystic ovarian syndrome and any other endocrine disorder; no natural luteal phase prior to treatment cycle; abnormal uterine cavity as evaluated by ultrasonography; presence of a clinically significant systemic disease.

### 2.2. Ovarian Stimulation Protocol and Evaluation Parameters

Patients were treated in a standard long GnRH-agonist protocol with the following sequence. GnRH-agonist, Triptorelin (Diphereline), was injected in the midluteal phase (day 21) at the dose of 1.0–1.875 mg and the pituitary down-regulation effect was confirmed by serum FSH (<5 IU/L), LH (<5 IU/L) and E2 (<50 pg/mL) at least 12 days after the injection, as well as transvaginal ultrasound assessment of follicles size (<10 mm) and endometrial thickness (<5 mm).

After pituitary down-regulation, the ovarian stimulation was initiated with human recombined FSH (rFSH, Gonal F or Puregon) using an individualized dose of between 100 IU and 375 IU i.m. per day according to age, ovarian volume, baseline FSH, and BMI. The ovarian response was monitored by ultrasound examination and serum LH, E2 and progesterone (P4) from days 6–8 of stimulation, and the dose of FSH administered was adjusted with (*n* = 94)/without (*n* = 28) 75 IU HMG (Urofollitropin for Injection-Livzon Pharmaceutical Factory) adding into the protocol. Since every 75 IU HMG contains activities of 75 IU of FSH, 75 IU of rFSH was cut down from patients who were supplied with HMG in order to balance the dosage.

When at least two follicles had reached a diameter of ≥18 mm, 10,000 IU of human chorionic gonadotropin (HCG) was administered to induce final follicular maturation. Oocyte retrieval was performed 36 h later by transvaginal ultrasound-guided follicle aspiration. Oocytes were cultured individually from the time of retrieval until the assessment on day 3 of after transfer. The fertilization rate was defined as the total number of fertilized oocytes divided by the total number of oocytes retrieved. A top quality embryo was characterized by the presence of 4-5 blastomeres on day 2 and 7-8 blastomeres on day 3, the absence of multinucleated blastomeres, and <10% cellular fragments. The top quality embryo formation rate was defined as the total number of top quality embryos divided by the total number of live embryos on day 3.

 A maximum of two embryos was transferred on day 3 after retrieval and luteal phase support was given by daily injections of progesterone started from the day of oocyte retrieval continuing till the day of pregnancy test. A positive pregnancy test was defined by a plasma *β*-HCG concentration >10 IU/L. Implantation rate is defined as the total number of gestational sacs divided by the total number of embryos transferred. A clinical pregnancy was defined as an intrauterine gestational sac with a heartbeat 30 days after embryo transfer. 

### 2.3. Blood Samples and Hormone Assays

 Blood samples were taken on days 2–5 of natural menstrual cycle before any treatment and were taken on stimulation days 1 and 6–8 day of HCG. Serum samples were immediately analyzed for FSH, LH, E2, and P4 by electrochemiluminescence (ECL) immunoassay.

### 2.4. Statistical Methods

Due to changes in practice and the nonrandomized treatment schedules, the data could only be compared within databases based on the discriminating LH value. Hormone concentrations were compared using the *t-*test, and group comparisons were made using contingency table analysis. Data were deemed to be significantly different when *P* ≤ 0.05. Computation was performed using SPSS statistical software version 12.0.1 for Windows. 

## 3. Results

### 3.1. Demographics

 A total of 122 normogonadotropic infertile patients were enrolled in the study. 28 of these patients received rFSH stimulation alone, while the other 94 patients received both rFSH and HMG. Patients' distribution and demographic characteristics are summarized in Table 1. There is no significant difference in the basic clinical features between the two groups of patients.

 Causes of infertility in the patient population are also depicted in Table 1. Proportions of couples with solely female or combined male infertility were similar between the groups. 

### 3.2. Safety Results

 Treatment with HMG was well tolerated. There were no serious or significant adverse events during the study. However, HMG may increase the risk of ovarian hyperstimulation syndrome (OHSS) and cause a higher cancellation rate in the fresh transfer cycles. Out of the 94 patients who received HMG supplement, 14 fresh transfer cycles were cancelled for being at risk of OHSS, but none was cancelled in the rFSH alone group.

### 3.3. Outcomes of the Controlled Ovarian Hyperstimulation

There was no significant difference neither in the pregnancy rate (71.4% versus 66.3%, *P* > 0.05) nor in the number of oocytes retrieved, number of matured oocytes, top quality embryo formation rate, or other related indicators of pregnancy outcome (Table 3). The gonadotropin treatment duration was not shortened by LH administration and the total dose of gonadotropin was similar between the groups. However, there was a higher level of serum oestradiol (E2) on the day of human chorionic gonadotrophin (HCG) (1999.10 ± 860.50 IU/L versus 2883.29 ± 1427.382 IU/L, *P* = 0.000) but lower fertilization rate (89.1% versus 69.6%, *P* < 0.000) in patients getting HMG supplementation (Table 2). 

## 4. Discussion

As the threshold theory of LH in ovarian function advocates, the ovarian follicle requires a minimal amount of LH for steroidogenesis (<1% of receptors attached by LH), meanwhile excessive stimulation of the ovaries by LH may adversely affect human preovulatory follicle development [3, 8, 9]. A retrospective analysis [7] in 2000 showed that normogonadotrophic patients undergoing long GnRH-agonist protocol treatment were five times more likely to suffer from early pregnancy loss when midfollicular phase LH serum concentrations were below 0.5 IU/L. However, Kolibianakis et al. [10] identified exactly opposite results. Besides, in an opening study of Humaidan, the implantation rate of patients with mid-follicle phase LH ≥1.99 IU/L were significantly increased by LH supplementation [11], but he himself also identified a significantly reduced pregnancy outcome in patients with endogenous LH level >1.51 IU/L [12]. Although more and more attention has been paid to the LH concentrations during mid-follicle phase [10–13], there were still no consensus on the optimal amount of LH and the best timing of LH supplementation during follicle development. Therefore, we performed this study based on the clinical outcomes of our reproductive center aiming to find out the effect of LH (HMG) supplement when the mid-follicle phase LH level ranges in 0.5–1.5 IU/L. 

We found that adding HMG to an rFSH-driven stimulation in long GnRH-agonist protocol is associated with a significant increase in serum E2 on the day of HCG (Table 2). Since E2 levels were similar on stimulation days 6–8 in the two populations, it can be assumed that the higher serum E2 values in the study population can be attributed to HMG supplementation, which possibly exerts an effect under the circumstances of relative LH depletion caused by GnRH-agonist downregulation, but it must also be noted that the higher E2 serum levels in HMG supplement group might also be partially caused by the FSH/HCG activity in HMG, although we have cut down 75 IU of rFSH from those patients. There was no significant difference in the number of oocytes retrieved, but patients supplied with HMG seemed to have more preovulatory follicles which may be associated with the higher level of E2 and also correspond to the higher risk of OHSS (one of the most serious complications during ovarian stimulation).

Besides, the fertilization rate was significantly lower in patients who received HMG supplement, although there was no significant difference in the number of retrieved oocytes or other pregnancy-related parameters. As has been pointed out, depending on the stage of development, follicles exposed to inappropriately high concentrations of LH may enter atresia or become prematurely luteinized and oocyte development may be compromised [3]; we suppose that exogenous LH supplementation is not needed when serum LH concentration of the mid-follicle phase is around 0.5–1.5 IU/L which is enough for follicle development during the long GnRH-agonist protocol. However, since this was a retrospective analysis, the case number in each group was not balanced there may be a recall bias. Therefore, a prospective, random control study should be carried out in the further research.

## Figures and Tables

**Table 1:** Patient population baseline characteristics.

Baseline parameters	rFSH (*n* = 28)	rFSH/HMG (*n* = 94)	*P*
Age (years)	29.57 (±4.05)	30.06 (±3.97)	NS
Duration of infertility (years)	4.07 (±2.39)	4.59 (±3.10)	NS
Total antral follicle count	13.54 (±4.11)	14.00 (±4.18)	NS
Baseline FSH (IU/L)	6.64 (±1.35)	6.80 (±1.27)	NS
Baseline LH (IU/L)	4.66 (±1.36)	4.68 (±1.68)	NS
Baseline E2 (pg/mL)	35.35 (±11.97)	36.68 (±14.85)	NS
BMI	21.37 (±2.00)	20.76 (±2.07)	NS
LH on stimulation day 1 (IU/L)	1.32 (±0.80)	1.46 (±0.85)	NS

Cause of infertility			
Tubal factor only	19 (67.9%)	55 (58.5%)	NS
Endometriosis	1 (3.6%)	5 (5.3%)	NS
With male factors	8 (28.6%)	34 (36.2%)	NS

Values are mean ± SEM.

**Table 2:** The stimulation and response details.

Parameters	rFSH (*n* = 28)	rFSH/HMG (*n* = 94)	*P*
LH on stimulation day 6–8 (IU/L)	0.98 (±0.24)	1.07 (±0.26)	NS
E2 on stimulation day 6–8 (pg/mL)	979.20 (±863.65)	804.13 (±736.54)	NS
Duration of stimulation (days)	11.46 (±2.24)	11.47 (±2.32)	NS
Total dose of gonadotrophins (IU)	2280.36 (±772.15)	2072.47 (±613.20)	NS
E2 on HCG (pg/mL)	1999.10 (±863.50)	2883.29 (±1427.38)	0.000
LH on HCG (IU/L)	1.17 (±0.61)	1.49 (±0.88)	NS
Number of oocytes retrieved	11.55 (±5.33)	13.68 (±5.42)	NS

Values are mean ± SEM.

**Table 3:** Outcomes of this IVF/ICSI cycle.

Parameters	rFSH (*n* = 28)	rFSH/HMG (*n* = 94)	*P*
Fertilization rate (%)	89.1 (303/340)	69.6 (860/1236)	<0.000
Top quality embryo formation rate (%)	49.6 (125/252)	48.2 (366/760)	NS
Clinical pregnancy rate (%)	71.4 (20/28)	66.3 (53/80)	NS
Implantation rate (%)	40.7 (24/59)	43.2 (73/169)	NS
Early pregnancy loss rate (%)	10 (2/20)	5.7 (3/53)	NS

Values are mean ± SEM.
